# Bayesian spatiotemporal modeling with sliding windows to correct reporting delays for real-time dengue surveillance in Thailand

**DOI:** 10.1186/s12942-020-00199-0

**Published:** 2020-03-03

**Authors:** Chawarat Rotejanaprasert, Nattwut Ekapirat, Darin Areechokchai, Richard J. Maude

**Affiliations:** 1 Department of Tropical Hygiene, Faculty of Tropical Medicine, Mahidol University, Bangkok, Thailand; 2 Mahidol-Oxford Tropical Medicine Research Unit, Faculty of Tropical Medicine, Mahidol University, Bangkok, Thailand; 3 Bureau of Vector Borne Disease, Department of Disease Control, Ministry of Public Health, Nonthaburi, Thailand; 4 Harvard T.H. Chan School of Public Health, Harvard University, Cambridge, MA USA; 5 Nuffield Department of Medicine, Centre for Tropical Medicine and Global Health, University of Oxford, Oxford, UK

**Keywords:** Real time, Dengue, Surveillance, Delay, Bayesian, Spatiotemporal

## Abstract

**Background:**

The ability to produce timely and accurate estimation of dengue cases can significantly impact disease control programs. A key challenge for dengue control in Thailand is the systematic delay in reporting at different levels in the surveillance system. Efficient and reliable surveillance and notification systems are vital to monitor health outcome trends and early detection of disease outbreaks which vary in space and time.

**Methods:**

Predicting the trend in dengue cases in real-time is a challenging task in Thailand due to a combination of factors including reporting delays. We present decision support using a spatiotemporal nowcasting model which accounts for reporting delays in a Bayesian framework with sliding windows. A case study is presented to demonstrate the proposed nowcasting method using weekly dengue surveillance data in Bangkok at district level in 2010.

**Results:**

The overall real-time estimation accuracy was 70.69% with 59.05% and 79.59% accuracy during low and high seasons averaged across all weeks and districts. The results suggest the model was able to give a reasonable estimate of the true numbers of cases in the presence of delayed reports in the surveillance system. With sliding windows, models could also produce similar accuracy to estimation with the whole data.

**Conclusions:**

A persistent challenge for the statistical and epidemiological communities is to transform data into evidence-based knowledge that facilitates policy making about health improvements and disease control at the individual and population levels. Improving real-time estimation of infectious disease incidence is an important technical development. The effort in this work provides a template for nowcasting in practice to inform decision making for dengue control.

## Background

Dengue is a mosquito-borne infectious disease that causes a huge economic and public health burden in Thailand and other tropical countries. Across Thailand, approximately 100,000 annual cases are notified to the Bureau of Epidemiology, Thai Ministry of Public Health [1]. Dengue infection poses a major problem for public health officials in the country with epidemics occurring every 2 to 3 years. These can place a huge burden on public health infrastructure in an affected province. Dengue transmission in Thailand is seasonal, usually with a peak in the rainy period from May to October [2]. Bangkok, divided into 50 administrative districts, consistently has the highest number of cases in the country. Incidence data show dynamic patterns in space and time with large between-district variation. Reporting delays make quantifying and predicting the trends in dengue cases in real-time very difficult causing resource allocation for public health control measures to be an ongoing challenge [3–5]. Factors shown to be associated with the disease pattern in Bangkok include population density, infrastructure, mosquito density, environmental management, expansion of the city, and changing lifestyles [6–8].

Thailand initiated a passive surveillance reporting system for dengue in 1958 and the national surveillance system became fully operational in 1974 [9]. All cases meeting the case definition are required to be notified but there is no requirement for confirmation with a diagnostic test. A further weakness of the system is a reliance on the willingness of physicians to report cases. In Thailand, trained physicians use the WHO case definition established since the 1970s and updated in 1997 to diagnose dengue cases, which uses clinical signs and symptoms to categorize them into 3 classes, dengue fever (DF), dengue hemorrhagic fever (DHF) and dengue shock syndrome (DSS) [10]. Prior to 1999, reports of dengue cases were sent by post; electronic transmission of reports began in 1999 [9]. For the past 10 years, the Thai surveillance system at the central level has been systematized and adopted electronic forms while there is no mandatory digital form at the local level and paper forms are widely used. Local hospitals procure their own systems for entering and transferring data. Combinations of hardcopy and electronic reports are then used to notify cases from local health facilities to provincial health offices and to the Bureau of Epidemiology, Department of Disease Control, where data on all reported cases are collected and analyzed.

Infectious disease control requires effective and prompt responses to unanticipated increases in disease burden i.e. outbreaks/epidemics. Inability to produce timely and accurate estimation of infectious disease burden can significantly impact public health control programs. A key challenge for dengue surveillance in Thailand, in common with many countries, is the delay before reports are received at different levels in the system at which they might trigger such a response. This results in an initial under-estimate of the true burden and a late, or no, response. Broadly, the delay may occur at any point in the time between the patient deciding to seek care and the case appearing in the surveillance database. Case reports have to go through several levels from local health facilities to the national surveillance center. In a particular year, approximately 75% of the dengue reporting delays in Thailand were up to 10 weeks [11].

Accounting for systematic delays in reporting has a historical precedent in actuarial sciences in modeling claims reserves [12, 13] and has been addressed for health outcomes in HIV/AIDS [14–16], mortality reporting [17] and for chronic diseases e.g. cancer registries [18]. Methods have also been developed in the context of infectious disease outbreaks [19–21]. Methods have been developed to nowcast, (i.e. estimate in real time) the current number of infected cases. Höhle and Heiden forecasted the daily hospitalized number of cases of hemolytic uremic syndrome [19]. Noufaily et al. proposed a method for detection of infectious disease outbreaks from laboratory data with reporting delays [20] based on a quasi-Poisson algorithm [22]. The field of infectious disease modeling has a rich literature of methodologies but has not previously focused on the challenge of estimating spatiotemporal reporting delays in real-time public health control applications. An integrated dengue monitoring system was developed in the city of Rio de Janeiro, Brazil, which corrects for reporting delays using a lognormal survival model (https://info.dengue.mat.br/informacoes/) [23]. The model has been further developed and is being used as a decision making tool by Brazilian authorities as warning systems, infoDengue (https://info.dengue.mat.br) and infoGripe (http://info.gripe.fiocruz.br) [24].

Monitoring and accounting for timeliness is essential for detecting dengue outbreaks that demand immediate public health responses and vector control measures. Efficient and reliable surveillance and notification systems are vital to monitor health outcome trends and early detection of disease outbreaks which vary in space and time. An important feature of spatiotemporal surveillance models is that the cumulative amount of data and associated parameters will increase as the system continues. As new data arrive, the estimation of the model in a surveillance context can be relatively slow. One approach to make the computation feasible for real time applications is particle filtration, which resamples the past realizations from the posterior distribution to give reweighted estimates [25]. However, this method critically depends on sufficient samples at the initial surveillance process to allow for efficient subsequent resample. Otherwise, the ability of the samples to cover the posterior distribution may degenerate overtime [26]. An alternative approach is the sliding window method. This mechanism, also known as batch processing, has been widely used in public health applications [27, 28]. In the sliding window method, a window of specified length moves over the data and the parameters are estimated by the most recent data with fixed time units [29, 30] as the necessary information only contained in the most recent data resulting in redundancy reduction in computing of a surveillance system.

In this work, we present a decision-support tool using a spatiotemporal nowcasting model which accounts for reporting delays in a Bayesian framework with sliding windows. A user interface was designed so this tool could be used by the dengue control program. This tool provides the program with more accurate and timely disease burden estimates which inform better planning of public health interventions for dengue control. Producing accurate and actionable estimations of incidence in real time will particularly enhance public health responses to outbreaks. A case study is presented to demonstrate the proposed nowcasting method and tool which accounts for delays of dengue surveillance in Bangkok in 2010.

## Methods

### Dengue surveillance data

**Fig. 1:**
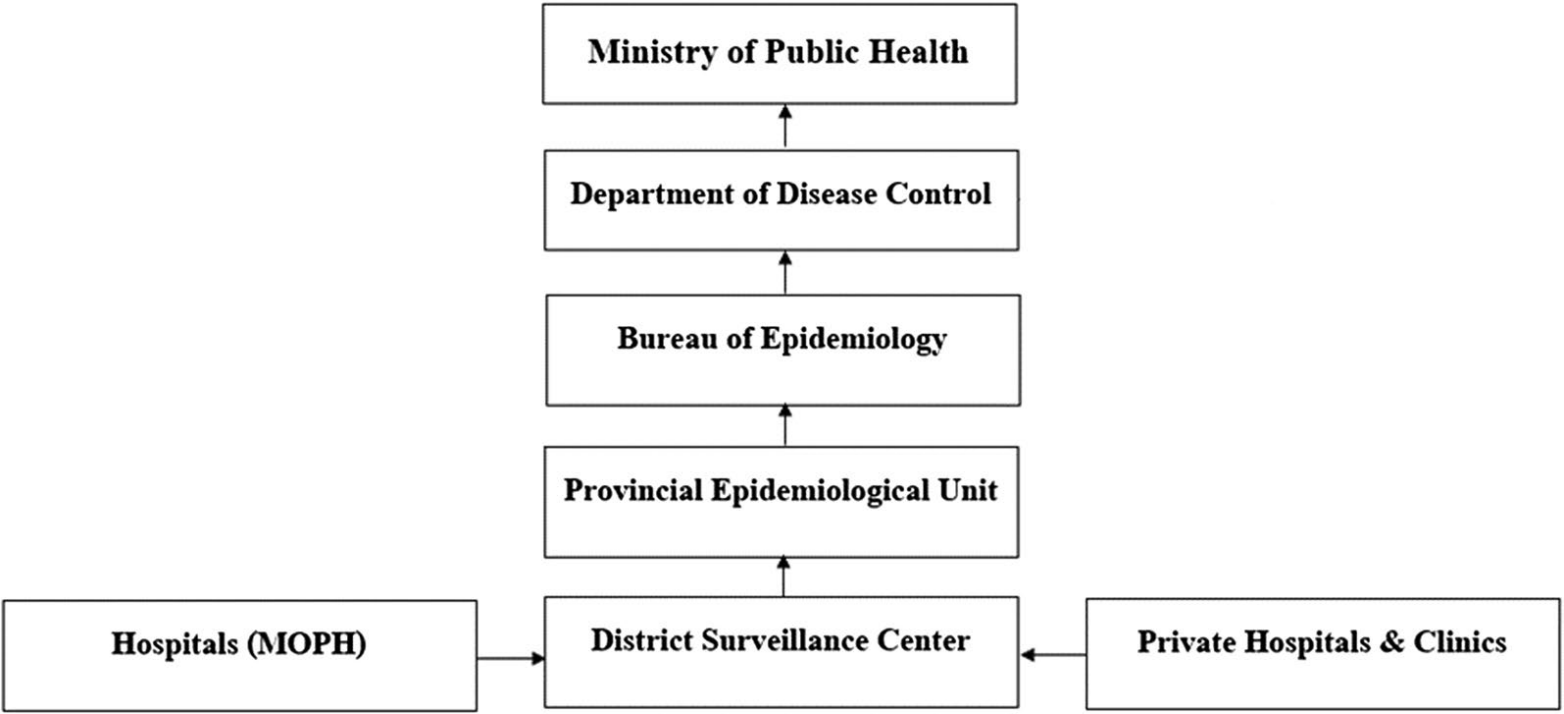
Diagram of the dengue surveillance reporting system in Thailand. Arrows represent flows of individual case data

Data on reported dengue cases were collated from the routine surveillance system of the Bureau of Epidemiology, Ministry of Public Health, Thailand. The surveillance data consisted of indigenous and imported cases that were collected from hospitals and clinics under the government universal health coverage schemes plus diagnosed cases from private hospitals, all of which are reported to district health surveillance information centers (Fig. 1). Reporting delay was defined the time in days between individual cases being diagnosed and entering the national surveillance database.

### Dengue nowcasting corrected for reporting delay

**Fig. 2:**
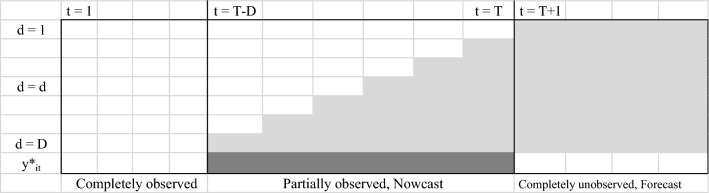
Real-time surveillance reporting delay structure. The white cells represent available cases in the system for each district at week *t* and reporting delays are in light grey cells. The dark grey represents the nowcasting of dengue cases accounting for reporting delays, $y_{it}^{*}$. *d* is the delay index with *D* maximum delays, i.e., delays beyond *D* are not considered. *t* is the time index and *T* indicates the current time point

The data structure of surveillance data with reporting delays can be viewed as the delay triangle depicted in Fig. 2. As described in [24], let $y_{itd}$ be the number of dengue cases which happen at district *i* (*i *=1,…, *I*) and calendar week *t* (*t* = 1,…, *T*) but appear in the surveillance database *d* (*d* = 1,…, *D*) weeks after the onset date. This represents the issue that cases have occurred but have not yet been reported. Note that *d *= 1 means that the case was in the surveillance database in the same week as the date of diagnosis. In Fig. 2, the subscript *t* = T indicates the current time point of interest and *D* is the maximum possible delay that can happen in the surveillance system, i.e. full data are available in the system from *T *+ *D* weeks. Let $y_{it}^{*} = \sum\nolimits_{d = 1}^{D} {y_{itd} }$ be the number of actual cases by adding estimated (occurred but not yet reported) delay fractions occurring at week *t*, $y_{itd}$, over the possible delay range. The aim of this study was to estimate the actual weekly number of dengue cases for each district, $y_{it}^{*}$, in a real-time manner for facilitating public health practitioners to anticipate outbreaks and plan disease control activities.

In this study, data on individual dengue cases from the 50 districts in Bangkok in 2010 were anonymized to protect their identity and then aggregated to weekly intervals and by district. The reported types of dengue were dengue fever, dengue hemorrhagic fever and dengue shock syndrome. For this study we focused on real time surveillance to control dengue infection and control activities can then be implemented as soon as possible to prevent disease transmission. Hence the combined number of dengue types was used in this situation.

Creating predictions in real-time poses data management and analytical challenges. Analytical challenges include model validation and adjustments for incomplete or delayed case reporting. Raw case information must be made available in a pre-processed format before analysis. In this work, raw data were aggregated to district level by week which is a timescale on which the disease control activities are implemented.

We assumed that the weekly district-level reported cases with delays followed a Poisson distribution where the offset, $n_{it}$, is assumed to be the number of population at risk in each district. There are multiple ways to calculate the offset and a commonly used method is the indirect standardization [31]. However, we do not know the number of true cases (without delays) at the time we analyze the data and we aim to estimate this from the model. Thus the population at risk is appropriate for the offset in this situation. Here the whole population in each district was assumed to be susceptible. The number of observed dengue cases (with delays) occurring within week *t* at district *i* is then represented as a random variable $y_{itd} \sim Poisson(n_{it} \theta_{itd} )$ where $\theta_{itd}$ is the relative dengue case risk adjusted for the number of population at risk. A main parameter of interest is $\theta_{itd}$ and the most common approach to model this is to assume a logarithmic link to a linear predictor.

The aim of this work is to estimate the number of dengue cases with reporting delays which can be considered as a latent variable. Thus a Bayesian framework consistent with the model and data generating process was adopted in which for all parameters in the model a prior distribution needs to be specified. A large literature on space–time modeling has been proposed based on Bayesian frameworks [31]. We structured the model by borrowing information across neighboring regions and time periods to incorporate spatiotemporal smoothing. A convolution model is specified to include delay, spatial, and temporal effects and their interaction random effects to capture structured and unstructured extra variation in the model.

In general, it is important to include both structured and unstructured random effects in spatial and spatiotemporal analyses because confounding can take various forms with both spatial and non-spatial structures. The unstructured random effect, $v_{i}$, is often described by a Gaussian prior distribution with zero mean and variance $\sigma_{v}^{2}$. The spatially structured effect, $u_{i}$, is assumed to follow the intrinsic conditional autoregressive (ICAR) model proposed by Besag et al. [32]. This model has a conditional form of $u_{i} |\varvec{u}_{ - i} \sim N\left( {\bar{u}_{{\varOmega_{i} }} ,{{\sigma_{u}^{2} } \mathord{\left/ {\vphantom {{\sigma_{u}^{2} } {n_{{\delta_{i} }} }}} \right. \kern-0pt} {n_{{\delta_{i} }} }}} \right)$ where $\varvec{u}_{ - i}$ is the vector containing the correlated effect of all except the *i*th district. $\varOmega_{i}$, $n_{{\delta_{i} }}$ and $\bar{u}_{{\delta_{i} }}$ are a set of the first-order spatial neighbors, cardinality and the average of the neighborhood of the *i* th district respectively.$\sigma_{u}^{2}$ is the spatial component variance. Notably there are a number of models that can be applied for these spatial random effects, including simultaneous autoregressive (SAR) or other geostatistical models. Among the models, conditional autoregressive (CAR) priors are probably the most common approach in areal disease mapping. Various globally smoothed CAR priors have been developed, and a review and comparison can be seen in [33].

To capture the temporal patterns in dengue case and delay estimation, a random walk model is adopted. In general, a random walk is assumed to have a prior Gaussian distribution with mean as the previous time point which can be either positive or negative. Then the temporal trend can be expressed as $\lambda_{t} \sim N\left( {\lambda_{t - 1} ,\sigma_{\lambda }^{2} } \right)$ and $\eta_{d} \sim N\left( {\eta_{d - 1} ,\sigma_{d}^{2} } \right)$ which allows for a non-parametric type of temporal form. All random interaction terms, $\varsigma_{dt} ,\zeta_{it} ,\xi_{id} ,\psi_{itd}$, are specified by a Gaussian distribution with zero mean. With inclusion of covariates, the corresponding coefficients are often assumed to have a zero mean Gaussian prior. The covariance matrix can be modeled as independence or assume a structure if covariates are correlated. All precision (reciprocal of variance) parameters are described by Log-Gamma distribution with hyperparameters as 1 and 0.0005 for the CAR model and hyperparameters as 1 and 0.00005 for uncorrelated and random walk random effects. Hence incorporating this stochastic representation of covariates and random effects, our proposed model becomes:

1 $$\begin{aligned} y_{itd} \sim Poisson(n_{it} \theta_{itd} ) \hfill \\ \log (\theta_{itd} ) = \varvec{X}_{itd}^{T}\varvec{\beta}+ v_{i} + u_{i} + \lambda_{t} + \eta_{d} + \varsigma_{dt} + \zeta_{it} + \xi_{id} + \psi_{itd} . \hfill \\ \end{aligned}$$

We also considered a negative binomial (NB) base distribution with overdispersion parameter to capture the variability in estimation. This gives rise to $y_{itd} \sim Negtive\;Binomial(n_{it} \theta_{itd} ,\phi )$. Although the Poisson likelihood with random effects has been widely applied as a standard practice in Bayesian disease mapping [31, 34], assuming a negative binomial model can be appealing when the issue of overdispersion, i.e. its variance exceeds the mean, is evident relatively to the Poisson. Particularly, $E(y_{itd} ) = \mu_{itd} = n_{it} \theta_{itd}$ is the conditional mean of the negative binomial base distribution and $Var(y_{itd} ) = \mu_{itd} (1 + \mu_{itd} /\phi )$. The quantity $1/\phi$ is an overdispersion parameter and as $1/\phi \to 0$, the negative binomial converges to a Poisson distribution which corresponds to no overdispersion. Therefore, for highly dispersed count data the negative binomial model adds flexibility in accommodating heterogeneity which could yield improved model fit. So, we applied both Poisson and Negative Binomial models to the data and compared the results using accuracy calculated by the proportion of weeks that the real case numbers were contained in the predictive band.

One necessary feature of real time surveillance systems is computational feasibility. In some situations, using all the cumulative data may be redundant and only the most recent data may be relevant to capture information needed for disease monitoring. Hence the use of data partition such as sliding windows can be an alternative optimization technique to increase computational efficiency of the system. The sliding window process in our surveillance setting is equivalent to working with the likelihood function given by.

2 $$\begin{aligned} & \prod\limits_{t = 1}^{T} {\prod\limits_{d = 1}^{D} {\prod\limits_{i = 1}^{I} {f(y_{itd} |\theta_{itd} )} } } \times 1_{T - w + 1 \le t \le T} \\ & \quad= \prod\limits_{t = T - w + 1}^{T} {\prod\limits_{d = 1}^{D} {\prod\limits_{i = 1}^{I} {f(y_{itd} |\theta_{itd} )} } } \end{aligned}$$

where $1_{(.)}$ is an indicator function and *w* is the length of sliding window. Given a time series of weekly dengue cases, the sliding window technique examines the *w* most recent weeks and the window moves as the new data comes in. This technique has an advantage that it does not need to use the full data. Especially for real time surveillance purposes, the number of parameters can grow dramatically which might not be computationally practical in the long term.

As shown in the real time surveillance data triangle in Fig. 2, a number of dengue cases have occurred but are not yet reported into the surveillance system each week which is denoted as NA in the light grey cells. To estimate the true number of cases, the weekly under reported cases due to delays for each district need to be approximated. From a Bayesian perspective, the delays can be viewed as missing data and an approach to impute the missing is to apply the posterior predictive distribution which can compute both point estimates and associated uncertainty. The posterior predictive distribution of delays is in the form:

3 $$p(y_{itd}^{{}} |\varvec{y}_{w} ) = \int_{\varTheta } {p(y_{itd}^{{}} |\varTheta_{w} ,\varvec{y}_{w} )} p(\varTheta_{w} |\varvec{y}_{w} )d\varTheta_{w}$$

Where $\varvec{y}_{w}$ and $\varTheta_{w}$ denotes the data and parameters in the sliding window with length *w* used to fit the model.

Estimates from the posterior predictive distribution can be computed from converged posterior samples using sampling-based algorithms such as Markov chain Monte Carlo (McMC). However, timeliness is an important feature of real time estimation in infectious disease surveillance. With multi-dimensional model set up and cumulative surveillance data over time, the parameter space can expand quickly and demand computational resources exponentially. A more efficient approach to infer parameters in this context is the integrated nested Laplace approximation (INLA) [35]. With optimized numerical routines for performing the above computations and compatibility of our proposed model with INLA format, the proposed model was implemented using the numerical Laplace approximation in the R-INLA package available from www.r-inla.org.

The inla() function allows for missing values in the response variable, and computes the posterior marginal for the corresponding linear predictor. One does not need a posterior predictive simulation like in McMC approaches [36]. INLA will automatically compute the predictive distributions for all missing values in the response, which should be assigned a “NA” value when defining the data. More information about INLA and the code for implemented models are presented in the appendix. A graphical user interface implemented using R Shiny [37] was also developed to provide model accessibility for public health workers.

## Results

**Fig. 3:**
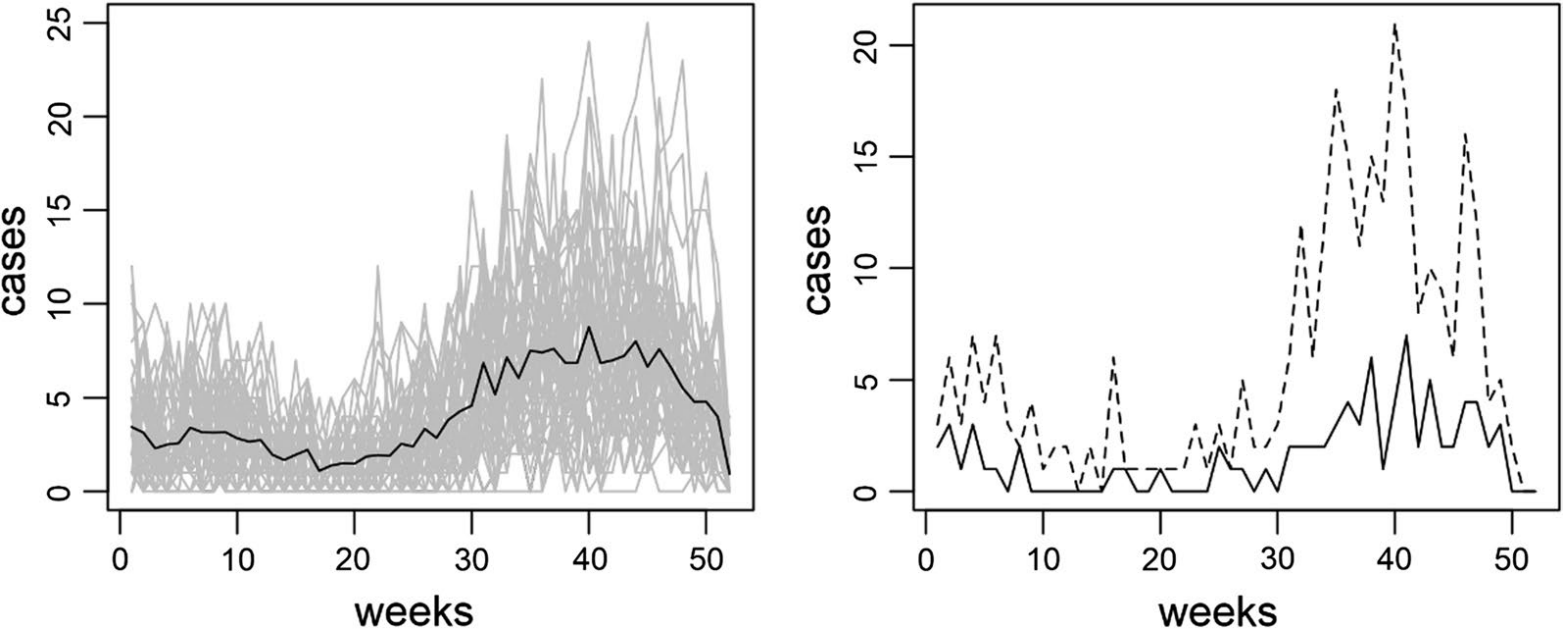
The left plot represents weekly dengue cases (grey) in Bangkok districts in 2010 with the mean averaged over all districts (solid). The right plot shows the actual weekly dengue cases (dash) and reported cases with delays (solid) in Bueng Grum district, Bangkok in 2010

**Fig. 4:**
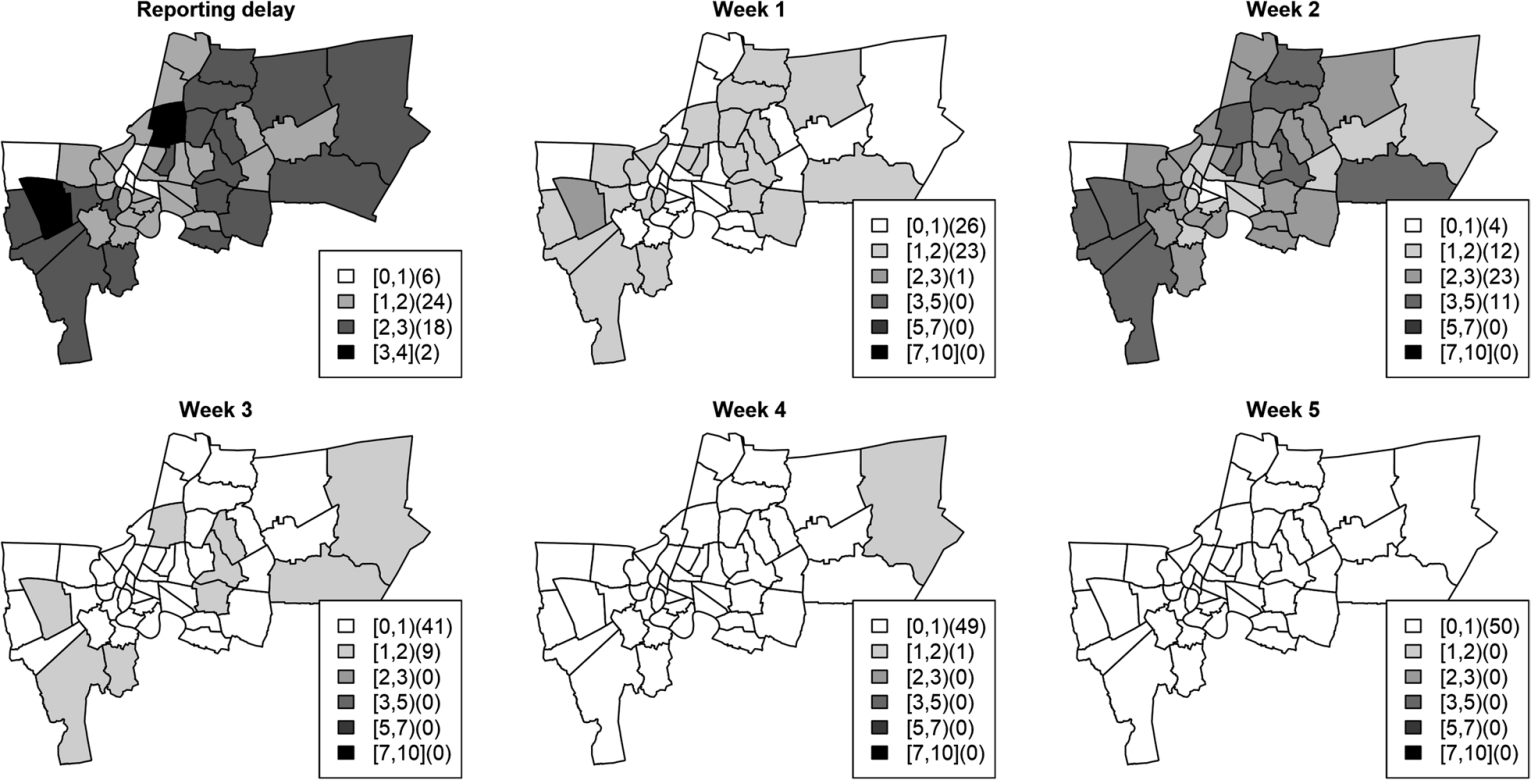
Maps of weekly reporting delays in each district. The reporting delay (upper left) map shows the average reporting delay in weeks across 52 districts in 2010. Panels week 1 to week 5 represent the average cases additionally reported 1–5 weeks after the onset date. The interval in square brackets represents the reporting delay or number of cases and the number in curved brackets represents the number of districts in that category

The available data used to demonstrate the proposed methodology were weekly counts of the number of dengue cases in the 50 districts in Bangkok during 2010 (Fig. 3). 77.24% of cases were reported late. 76.12% of reported cases were in the database by 2 weeks and almost all (98.7%) within 5 weeks. The mean and median reporting delay were 1.77 and 2 weeks respectively. In Bueng Grum district, a major endemic area in Bangkok, the reporting delays approached 20 cases during peaks in incidence (Fig. 3). The numbers in weeks of under reported dengue cases due to reporting delays by district averaged across 52 weeks in 2010 are shown in Fig. 4 (upper left panel). Most dengue cases were reported in the system after 2 weeks. This reporting gap is sufficient for further dengue transmission to occur, potentially making control activities more difficult.

Table 1 presents accuracy and computing time for Poisson and Negative Binomial models with different sliding window sizes. These comparisons were made on a Dell computer with 64 bit Windows system, 8 GB RAM and Intel i5-3570S CPU @ 3.10 GHz. The posterior summary of dispersion parameter ($1/\phi$) with its corresponding credible intervals (CrI) indicates a mild overdispersion in the data with posterior means of 0.03–0.09. This implies that the Poisson specification with random effects might be sufficient to capture the variability in the data set. To focus on operational practicality, accuracy and computing times were compared between Poisson and Negative Binomial models with different values of *w*. The Poisson model outperformed the Negative Binomial model across window sizes with respect to both accuracy and computing time. There is a computational cost for additional window size increase, but the use of historical data between 25–35 periods back seems to yield similar accuracy to the whole data set under both likelihood assumptions. Since the Poisson model appears to outperform the Negative Binomial with slight indication of overdispersion, the only results under Poisson likelihood are presented thereafter.

**Table 1:** Evaluation meausures for Poisson and Negative Binomial models with different sliding window sizes

*W*	Poisson		Negative Binomial		
	Accuracy	Time (min)	Accuracy	Time (min)	Dispersion (95% CrI)
10	0.72	1.57	0.61	2.81	0.03 (0.011, 0.091)
15	0.74	3.37	0.62	5.81	0.07 (0.046, 0.109)
20	0.77	6.06	0.67	10.86	0.08 (0.059, 0.123)
25	0.78	9.73	0.70	17.36	0.08 (0.051, 0.123)
30	0.79	14.39	0.73	25.52	0.09 (0.061, 0.135)
35	0.79	20.48	0.74	37.16	0.08 (0.051, 0.122)
Full data	0.80	42.23	0.75	62.91	0.08 (0.056, 0.131)

**Fig. 5:**
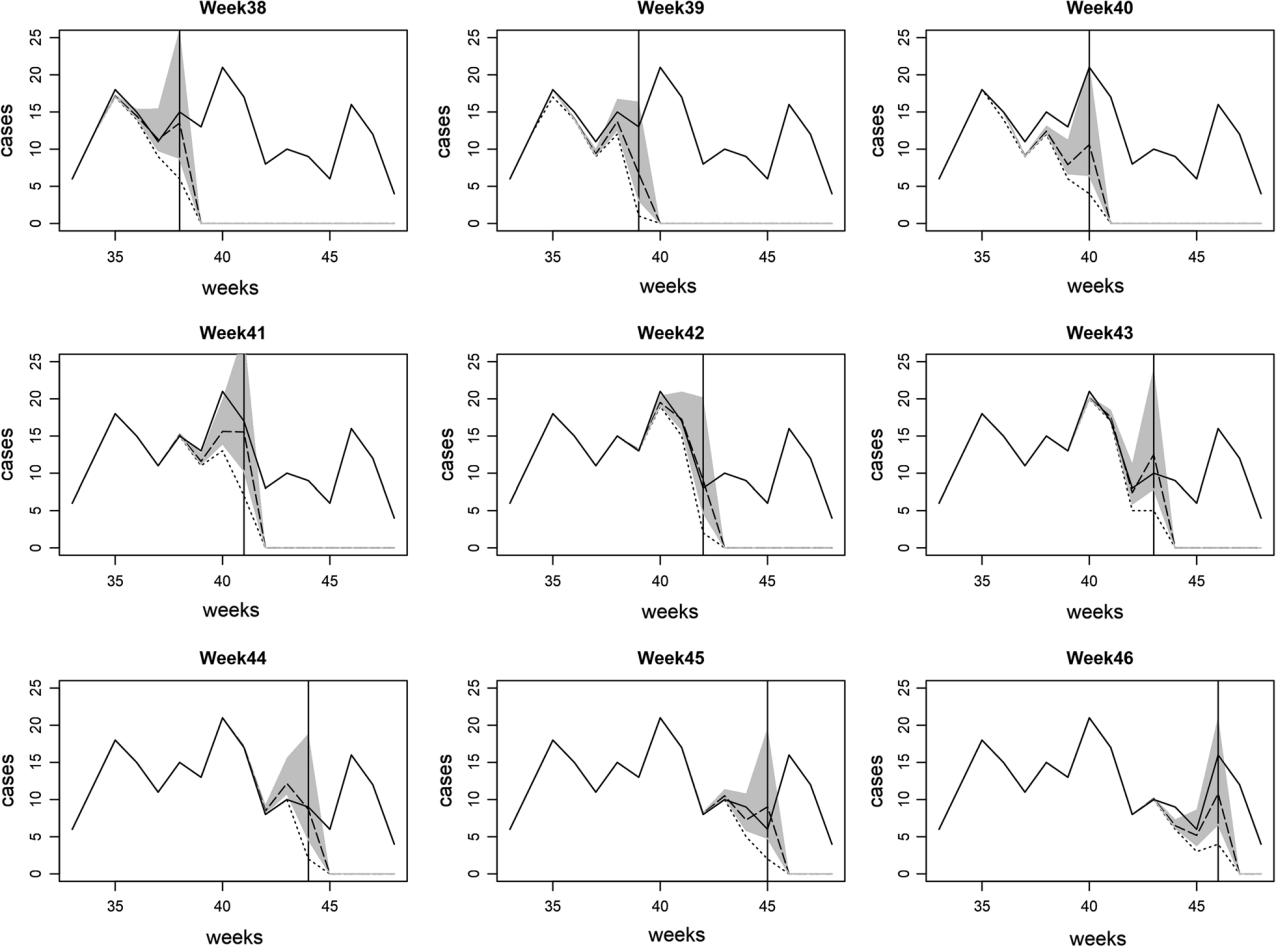
Comparison of numbers of cases with delay, delay corrected and true cases during weeks 38–46 in Bueng Grum district, Bangkok in year 2010. The real time estimation corrected for delays (dashed) under Poisson likelihood with full data is shown with corresponding 95% credible band. The solid and dotted lines are true cases and cases with reporting delays

**Fig. 6:**
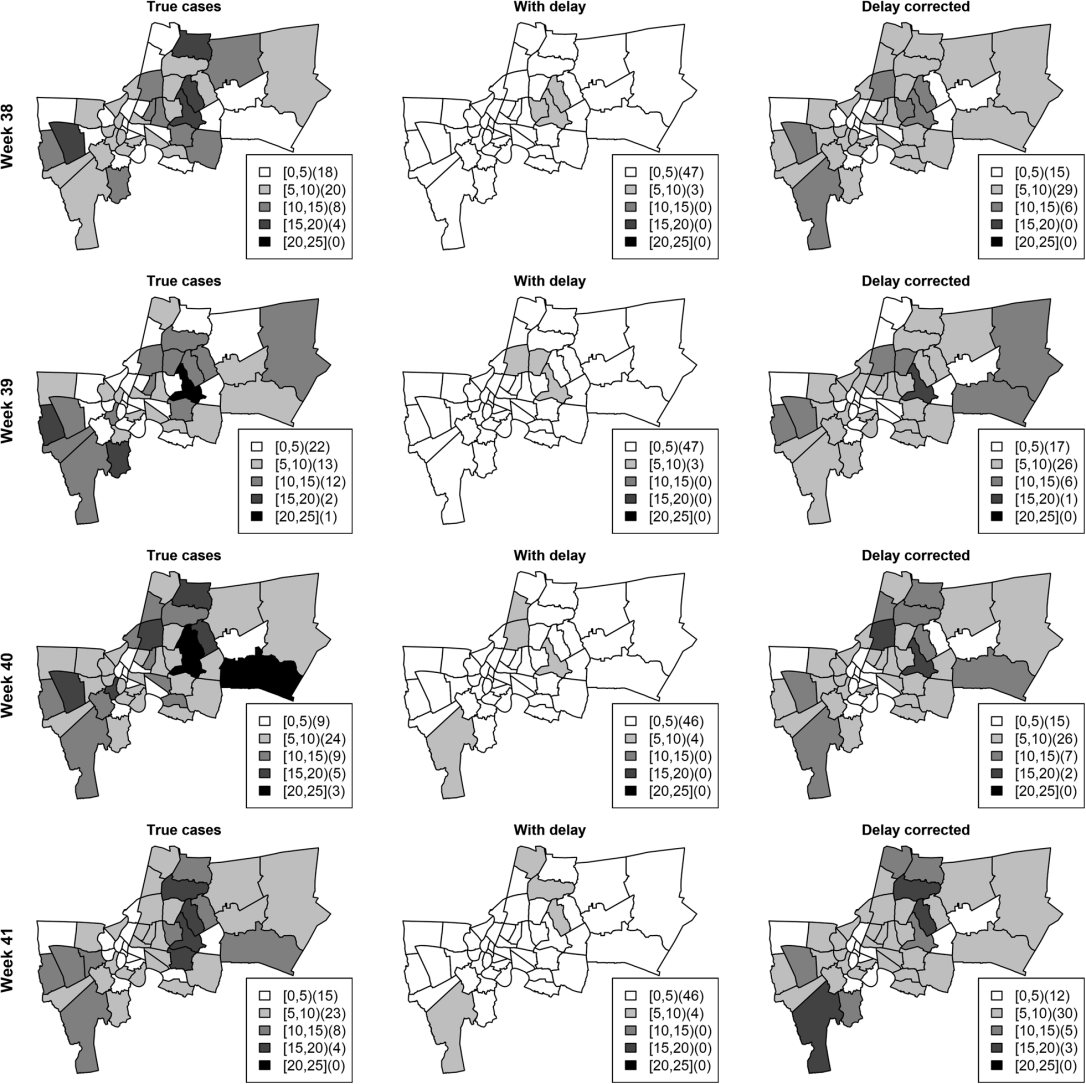
Comparison of true numbers of cases, cases with delay, and with delay corrected under Poisson likelihood with full data during weeks 39–41 for Bangkok districts in the year 2010

Our goal is to use the proposed model to correct reporting delays considering spatial and temporal variability in the delay mechanism. The proposed delay model was implemented without covariates and the true cases were estimated using their predictive posterior distribution. Figure 5 depicts the numbers of cases with delay, delay corrected and true cases during the high season of weeks 38–46 in Bueng Grum district, Bangkok in 2010. The model was able to estimate the true number of dengue cases within its credible band even during peaks. For example, there was a marked increase at week 40 with delay in reporting of almost 20 cases, but the model still could estimate the true case number included in the predictive interval. Figure 6 shows the maps of true cases, cases with delays (weekly observed), and corrected for delays during weeks 38–41 of all districts. The notified number of dengue cases may not represent the transmission intensity due to reporting delays. However it can be seen that the method gives a reasonable estimate of true numbers of cases at all these timepoints and facilitate the public health workers to implement control activities more effectively.

**Fig. 7:**
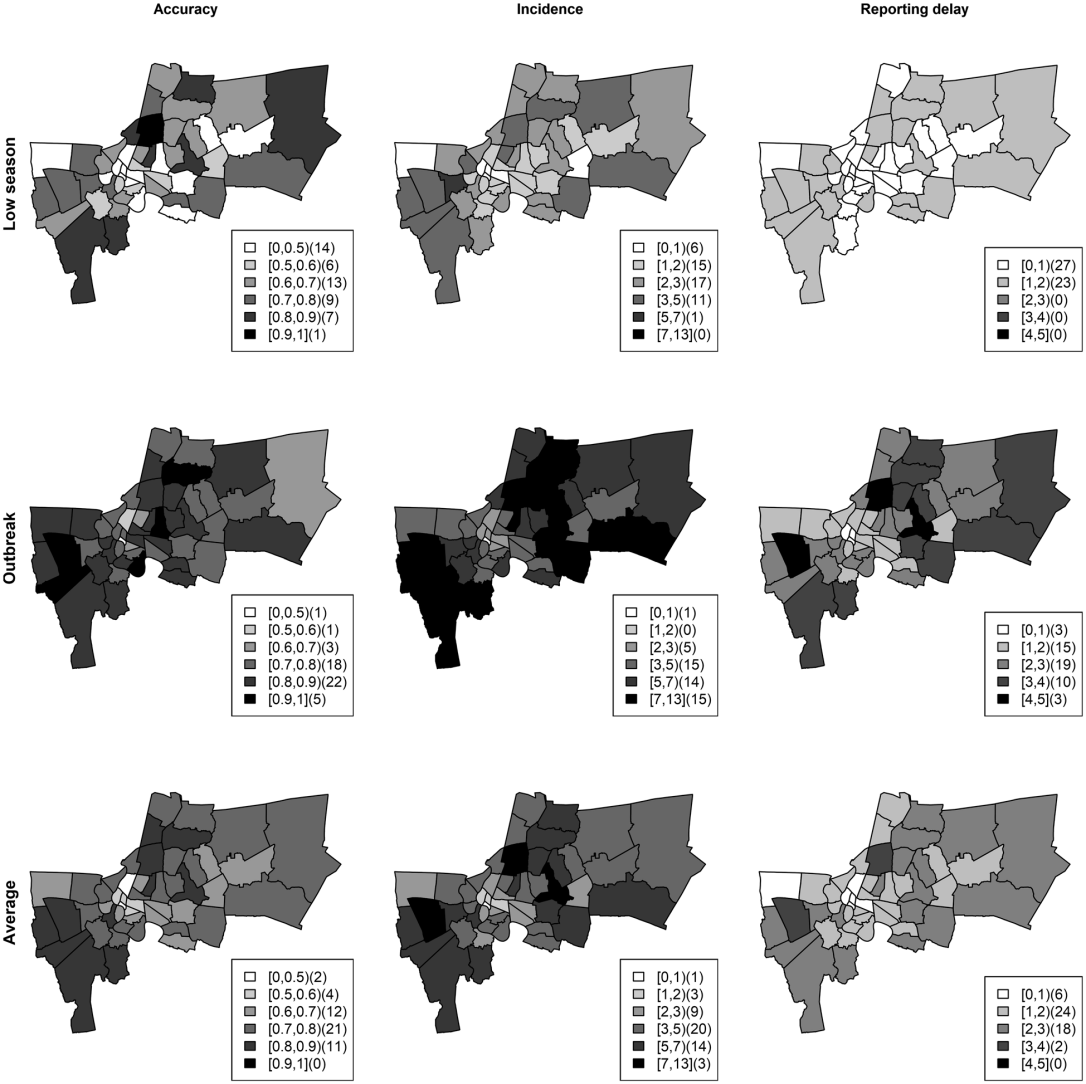
The accuracy under Poisson likelihood with full data using full data (proportion of weeks corrected cases in credible band), incidence and numbers in weeks of under reported cases during the low season (upper row), outbreak (middle row) and averaged across the weeks in 2010 (lower row)

The accuracy calculated by the proportion of weeks that the real case numbers were contained in the credible band was 79.59% averaged across all districts. Most districts had 70–90% (Fig. 7, middle row) accuracy during the high season (weeks 26–52; Fig. 3, left plot). The areas with high accuracy corresponded to those which had high incidence and high numbers of under reported cases. This suggests that our model can recover the under reported portion due to systematic delays which has important implications for public health preparation for disease control during outbreaks.

To further evaluate the model performance, we also considered during the low season, (weeks 1–25, Fig. 3, left plot) and the accuracy was 59.05% averaged across districts. The true dengue incidence for each district is shown in the middle column of Fig. 7. The model appeared to work generally well in areas with high standardized incidence rate (SIR) and large numbers of delayed reported cases (Fig. 7, upper row). However, the overall accuracy was 70.96% and most districts had accuracy between 70-90% averaged across all weeks (Fig. 7, lower row). The model in general was able to estimate the numbers of true cases in the presence of delayed reports in the surveillance system.

**Fig. 8:**
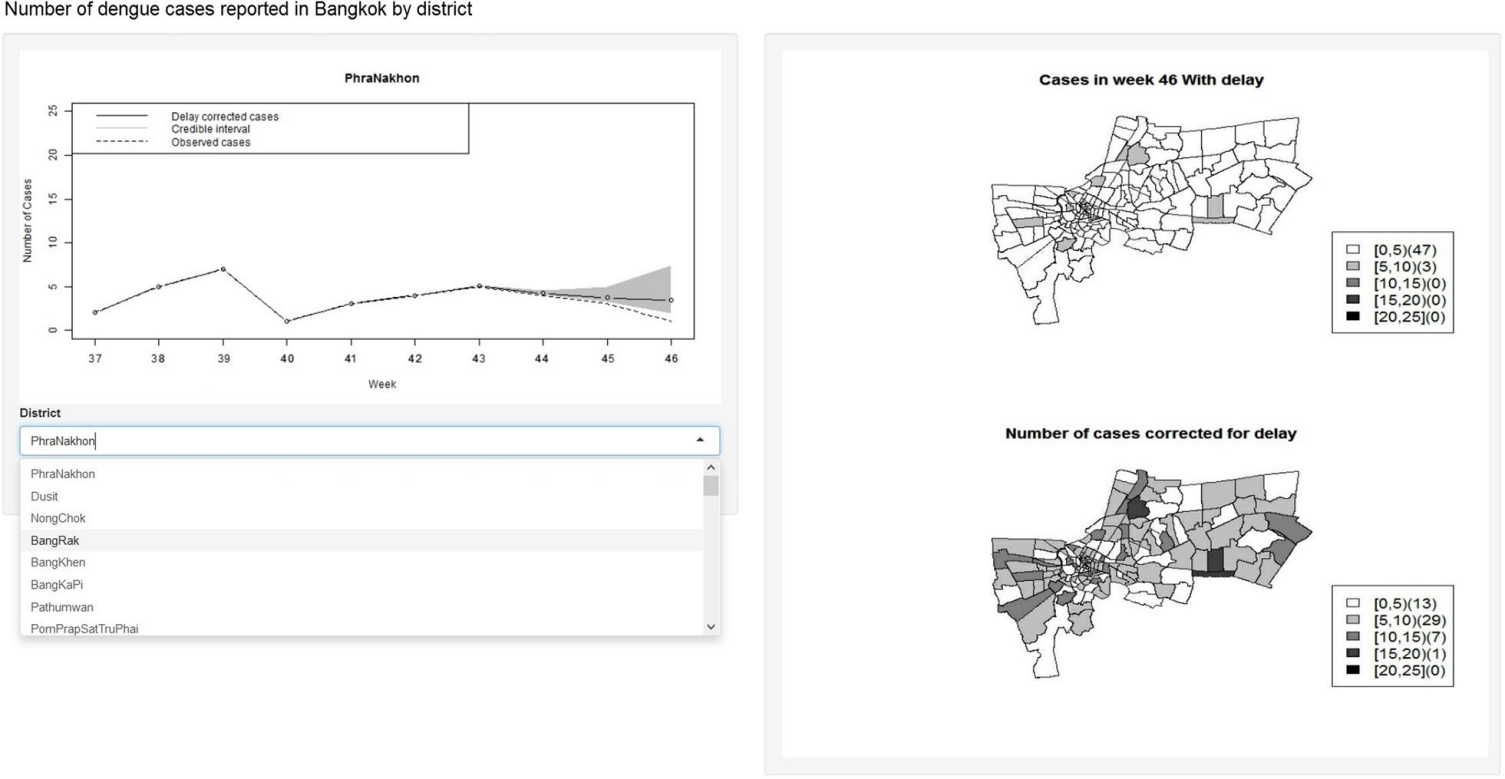
Screenshot of the visualization interface featuring the nowcasting model. On the left panel, the weekly dengue cases accounted for reported delays is depicted as a solid black line with 95% credible band in grey, the dashed line represents the observed cases in a Bangkok district which is subject to systematic delays. A dropdown is provided under the plot for users to specify the district. Maps on the right panel represent the weekly observed (upper map) and estimated (lower map) numbers of cases in all Bangkok districts

To provide accessibility of the proposed model, an interactive tool was developed in collaboration with the national dengue control program, Ministry of Public Health, Thailand for use by public health workers to apply the same delay reporting correction using RShiny from Rstudio [37], with a screenshot shown in Fig. 8. The tool allows filtering by district of the numbers of cases over time, as well as maps of delayed cases and corrected cases.

## Discussion

We present the nowcasting results from a real-time correction model for dengue surveillance in Bangkok, Thailand. This study addresses the impact of reporting delays on numbers of cases, a problem which is widespread in the surveillance of communicable diseases [11, 20, 21]. These systematic delays in the surveillance system can produce a big lag in case reports which lead to initial under-estimates of disease burden impacting on the planning and allocation of resource for disease control interventions and consequently suboptimal control with an impact on disease transmission and impairment of ability to efficiently tackle outbreaks. Reporting delays can be particularly problematic in more remote areas, where electronic surveillance forms are not mandatory with data having to go through several levels to reach the national surveillance database.

The infectious disease modeling literature has a rich and varied selection of transmission modeling but has not previously focused on the challenge of estimating spatiotemporal reporting delays in real-time situations. A spatiotemporal predictive model was developed to account for reporting delays in a Bayesian framework with sliding windows. Two model variations under Poisson and Negative Binomial likelihoods were examined with the assumption of possible overdispersion. The posterior summary of the dispersion parameter in Negative Binomial base distribution indicates a mild overdispersion in the data. The Poisson model also outperformed the Negative Binomial model across the evaluation metrics. The results suggest that the standard practice using Poisson likelihood with random effects might be sufficient to capture extra variation in our spatiotemporal surveillance data. This might be because the Poisson model offers a flexible posterior distribution on the random effects, although a Normal distribution in the prior can have a different distribution in the posterior and so sufficiently capture the extra variation in the data well. However, variants of model specifications could be further explored under other dispersion distributions.

Here we also focused on practicality in real time applications and therefore proposed to implement the developed methodology using numerical integration opposed to conventional sampling-based approaches with different sliding window sizes to increase computational feasibility. This is a trade-off between computational cost and accuracy of the model. The more we increse window size, the more we gain accuracy and also increase the comuting time. However, for the Poisson model, the accuracy increases by 8% from window size *w *= 10 weeks to using the full data while the computing time increase almost 30 folds. In addition if we had a long historical data, it would take cumulatively more time to compute where as the computing time is fixed for sliding windows with potentially similar predictive accuracy. So for real-time surveillaance the model can be fitted with a relative small sliding window size to obtain an initial analysis and a larger window size or full data can then be performed to confirm later. Hence the use of data partiton such as sliding window, which prevents refitting the whole historical data and reduces comptational time, might be useful and should be considered in real time surveillance applicaitions.

Our nowcasting in the case study varied by transmission intensity and location in quality and public health utility. The average delays were quite small during the low transmission period. The model might produce over estimation due to the model assumption of underlying delays when in fact there was a low amount of delays especially during no outbreaks. However, the average accuracy when an outbreak occurred, which was the main time of interest for our study, was almost 80% and the overall predictive accuracy was in the range 70% to 90%. Most districts with good model performance also corresponded to those with high transmission and frequent delays. This suggests that our approach can provide important information to help with planning for outbreak control. The high season in this manuscript was defined as the rainy season which is associated with the number of dengue case in Thailand. Climatic or seasonal variables could also be included in the model. However, lagged effects should also be considered when quantifying the climatic or environmental association. Although climatic factors could be used to explain some of the temporal variation, a simple sliding window will make it very difficult to estimate. In addition, results in Table 1, where a reasonably large *w* gives the same performance as all of the data, would probably not be implied that is the case if we had several years of data and a seasonal effect in our model, as also suggested by Stoner and Economou in their work on delay reporting [38]. Nonetheless, the temporal random effect which was assumed as the random walk prior in this work might adequately capture temporal variation in the data set.

The simplicity of the methodology that we present in this work has both weaknesses and strengths. A major advantage is computational feasibility in real time surveillance. Compared the time taken to run our models to the arguably more complex work using McMC with NIMBLE presented in [38], which took 30-120 min to run just for time series data. Hence with a spatial dimension it could take a day or so to run, which may be impractical for real-world use. On the other hand, our implementation using INLA can be much faster and therefore more practical in an operational setting. Another advantage is that the data it uses are readily available to the dengue control program in Thailand and the methods could thus be applied in near real-time. This type of phenomenological spatiotemporal model tends to show good predictive performance in real-time situations over short timescales but may have deficiencies when long-term predictions are a focus. The method used in this work is based on a standard Bayesian spatiotemporal model. In fact, reformulation of the method to make the explicit connection between modeling auto-regressive disease counts in a disease transmission model context can be an enhancement and link to commonly used models in other fields. Model improvements under consideration include related environmental and socioeconomic covariates. However, such covariates are often not available quickly enough in the right format to include in timescales needed for planning during outbreaks.

Continued development and refinement of such nowcasting will enable existing dengue surveillance to reach its full potential in making an impact on public health decision-making and planning. Universal adoption of electronic reporting systems is likely to reduce reporting delays, but implementation would need considerable effort and investment across the various components of the health system.

## Conclusions

The past decade of biomedical and public health research has witnessed rapid progress in digital transition. A persistent challenge for the statistical and epidemiological communities is to transform data into evidence-based knowledge that facilitates policy making about health improvements and disease control at the individual and population levels. Improving real-time estimation of infectious disease is an important technical development. However, continued research and collaboration is needed to develop a better platform to integrate infectious disease modeling into public health practice. The effort in this work provides a template for nowcasting in practice to inform decision making for dengue control.

## Data Availability

The data that support the findings of this study were obtained from the Thai Bureau of Epidemiology, Ministry of Public Health, but restrictions apply to the availability of these data, which were used with permission for the current study, and are therefore not publicly available. However, data may be available from the authors upon reasonable request and with permission of the Thai Bureau of Epidemiology.

## Appendix

The integrated Laplace approximation (INLA) methodology was first introduced by Rue et al. [35], and is recently reviewed in Rue et al. [39]. It is a deterministic approach to approximate Bayesian inference for latent Gaussian models (LGMs). In most cases INLA is both faster and more accurate than MCMC alternatives for LGMs. An important consequence of the concept of exchangeability in Bayesian framework is that we can also derive a predictive result on the variable *Y*. In our application, the quantity of interest is $y_{it}^{*} = \sum\nolimits_{d} {y_{itd}^{{}} }$ representing the nowcasting of dengue cases accounting for reporting delays in area *i* (a value not yet observed) of the surveillance report at time period *t* described by the information gathered up to the current time period ***y***. If we assume exchangeability for the augmented dataset $\{ \varvec{y},y_{itd}^{{}} \}$ we then have as in [3] as.

$$p(y_{itd}^{{}} |\varvec{y}_{w} ) = \int_{\varTheta } {p(y_{itd}^{{}} |\varTheta_{w} ,\varvec{y}_{w} )} p(\varTheta_{w} |\varvec{y}_{w} )d\varTheta_{w}$$

where $\varvec{y}_{w}$ and $\varTheta_{w}$ denotes the data and parameters in the sliding window with length *w* used to fit the model.$p(y_{itd}^{{}} |\varvec{y}_{w} )$ known as predictive distribution, is only meaningful within the Bayesian approach, since the posterior distribution only exists if are the parameters of interest are random variables. The analytic form of the posterior predictive distribution in most spatiotemporal models is not available.

In conventional sampling-based Bayesian analysis, the prediction can be done by posterior predictive simulation, i.e., drawing random samples from the posterior distribution. In the INLA library, there is no function “predict” as, for example, *lm* command in R. However, one does not need a posterior predictive sampling like in McMC approaches [36]. Predictions can be done as a part of the model fitting itself in INLA. As prediction is the same as fitting a model with some missing data, we need to set the response variables “NA” for those observations” we want to obtain estimates from the predictive distribution [36, 40]. Suppose we have the real-time surveillance reporting delay structure as in Fig. 2, the estimated nowcasting corrected for reporting delays in our application can be implemented through the following R-INLA code:

#Set up the model.

f ←y ~ 1+f(ID.I ,model=“bym”,graph=dis.bkk.adj)+

f(ID.T,model=“rw1”)+f(ID.D,model=“rw1”)+f(ID.IT,model=“iid”)+f(ID.DT,model=“iid”)+f(ID.DI,model=“iid”)+f(ID.DIT,model=“iid”)

#Negative Binomial likelihood

model.nb ← inla(f,family= “nbinomial”, data=data, E=E,

control.compute=list(dic=TRUE,waic=TRUE,cpo=TRUE))

#Poisson likelihood

model.p ← inla(f,family= “poisson”, data=data, E=E,

control.compute=list(dic=TRUE,waic=TRUE,cpo=TRUE))

Suppose the surveillance data y follows the surveillance reporting delay structure with the observed case counts augmented with “NA” for those unobserved in each time period representing as in light grey cells in Fig. 2, the summary statistics of the predicted values can be accessed by the commands model.nb$summary.linear.predictor and model.p$summary.linear.predictor.

## Abbreviations

DF: Dengue fever
DHF: Dengue hemorrhagic fever
DSS: Dengue shock syndrome
SAR: Simultaneous autoregressive model
ICAR: Intrinsic conditional autoregressive
McMC: Markov chain Monte Carlo
INLA: Integrated nested Laplace approximation
LGM: Latent Gaussian model
